# High-dose radiation associated with improved survival in IDH-wildtype low-grade glioma

**DOI:** 10.1186/s41016-021-00239-z

**Published:** 2021-04-01

**Authors:** Shuai Liu, Yanwei Liu, Guanzhang Li, Jin Feng, Li Chen, Xiaoguang Qiu

**Affiliations:** 1 Department of Radiation Oncology, Beijing Tiantan Hospital, Capital Medical University, Beijing, 100070 China; 2 Department of Molecular Neuropathology, Beijing Neurosurgical Institute, Capital Medical University, Beijing, 100070 China

**Keywords:** Radiation dose, IDH-wildtype, Low-grade glioma, Survival

## Abstract

**Background:**

As molecular advances have deepened the knowledge on low-grade glioma (LGG), we investigated the effect of higher radiation dose on the survival of IDH-wildtype (IDHwt) LGG.

**Methods:**

In the current study, 52 IDHwt LGG patients who received radiotherapy were enrolled from the Chinese Glioma Genome Atlas dataset. Radiation doses > 54 Gy were defined as high-dose, whereas doses ≤ 54 Gy were defined as low-dose. We performed univariate and multivariate survival analyses to examine the prognostic role of high-dose radiotherapy.

**Results:**

In total, the radiation dose ranged from 48.6 Gy to 61.2 Gy, with a median of 55.8 Gy, and 31 patients were grouped into high-dose radiation. Univariate survival analysis indicated that high-dose radiotherapy (*p* = 0.015), tumors located in the frontal lobe (*p* = 0.009), and pathology of astrocytoma (*p* = 0.037) were significantly prognostic factors for overall survival. In multivariate survival analysis, high-dose radiotherapy (*p* = 0.028) and tumors located in the frontal lobe (*p* = 0.016) were independently associated with better overall survival.

**Conclusions:**

In conclusion, high-dose radiotherapy independently improved the survival of IDHwt LGG. This can guide treatments for glioma with known molecular characteristics.

## Background

Low-grade glioma (LGG) is a highly heterogeneous group of gliomas, mainly including astrocytoma and oligodendroglioma. The survival of LGG varies significantly, with fortunate cases reaching more than 10 years. However, some cases, like glioblastoma, are shortened [1, 2]. Since histopathological classification is insufficient to depict the biology of LGG, molecular advances play an important role. The 2016 World Health Organization classification of tumors of the central nervous system added molecular markers to histology in the classification system of gliomas [3]. In particular, the status of isocitrate dehydrogenase (IDH) mutation and codeletion of chromosome arms 1p and 19q (1p/19q codeletion) were identified biomarkers that yielded a more accurate diagnosis and prognosis for LGG.

Patients with IDH-wildtype (IDHwt) LGG had a significantly poor prognosis [1], and they benefitted from more aggressive treatments. Recent guidelines [4, 5] have identified IDH wildtype as a high-risk factor, and radiotherapy was suggested for these patients. However, the optimal radiation dose is still unclear. Two prospective clinical trials have revealed that increasing the radiation dose failed to prolong the survival of LGG patients [6, 7]. However, these results may be limited by the lack of molecular data. Since IDHwt is uncommon (less than 20% of all the LGGs [2, 8, 9]), there is still insufficient evidence on radiotherapy for this tumor.

In the current study, a cohort of IDHwt LGG was enrolled from the Chinese Glioma Genome Atlas (CGGA) dataset. Using univariate and multivariate survival analyses, we evaluated the prognostic role of radiation in IDHwt LGG. Our findings may improve the dismal prognosis of these tumors.

##  Methods

### Patients

In the current study, 52 patients were enrolled from the CGGA dataset (http://www.cgga.org.cn). The inclusion criteria were (1) newly diagnosed, pathology-confirmed diffuse glioma (WHO II); (2) age > 18 years; (3) received radiotherapy; and (4) possessed IDH mutation, radiation dose, and survival data. The current study was approved by the Ethics Committee of Beijing Tiantan Hospital, and written informed consent was obtained from all participants.

### Clinical data

Clinical information of all patients was obtained from the CGGA dataset. Age at diagnosis and preoperative Karnofsky Performance Status Scale (KPS) score were dichotomized as > 40 or ≤ 40 years, and as ≥ 70 or < 70, respectively. The extent of resection was evaluated by comparing the pre- and postoperative magnetic resonance images. Gross total resection (GTR) was defined as the removal of all abnormalities on T2/FLAIR-weighted images and failing to achieve GTR was defined as partial resection (< GTR). Seventeen (33%) patients received chemotherapy with carmustine, nimustine, or temozolomide.

### IDH mutation detection

The IDH1/2 mutation status was determined by the pyrosequencing method described in our previous work [10].

### Radiotherapy

Most patients (41, 79%) received 3D-conformal radiation therapy, and 11 patients received intensity-modulated radiation therapy (IMRT). A radiation dose > 54 Gy was defined as high-dose while a dose ≤ 54 Gy was defined as low-dose.

## Statistical analysis

Clinical characteristics were compared via the Chi-square test.

Progression-free survival (PFS) was calculated from the date of surgery to the date of disease progression, or date last known to be progression-free. Overall survival (OS) was from the date of surgery to the date of death or last follow-up, whichever occurred first. To evaluate the prognostic role of radiation, the Kaplan-Meier method was used and compared by log-rank test. Cox proportional hazards regression (backward stepwise) was performed to identify independent risk factors for survival. Statistical analysis was performed using R language (https://www.r-project.org/), and a probability value (*p*) < 0.05 was considered significant. Missing values were excluded from statistical analysis.

## Results

### Patient characteristics

Among the 52 patients, 37 were male (71%), and the median age was 42 years (range, 19-61 years). The median radiation dose was 55.8 Gy (range, 48.6. 61.2), and 31 patients were grouped into high-dose radiation. The comparison of clinical variables between the high- and low-dose radiation groups is shown in Table 1.

**Table 1:** Comparison of clinical characteristics

Characteristics	High-dose (%)	Low-dose (%)	***P*** value^a^
Number	31	21	
Age			**0.026**
> 40	14 (47)	16 (53)	
≤ 40	17 (77)	5 (23)	
Sex			0.509
Male	21 (57)	16 (43)	
Female	10 (67)	5 (33)	
Location			0.397
Frontal lobe	17 (65)	9 (35)	
Other	14 (54)	12 (46)	
Preoperative KPS^b^ score			
≥ 70	31 (60)	21 (40)	
< 70	0	0	
Histologic diagnosis			0.458^c^
Astrocytoma	13 (54)	11 (46)	
Oligodendroglioma	4 (67)	2 (33)	
Oligoastrocytoma	14 (64)	8 (36)	
Resection			**0.045**
GTR	16 (76)	5 (24)	
< GTR	15 (48)	16 (52)	
Chemotherapy			0.417
Yes	9 (53)	8 (47)	
No	22 (65)	12 (35)	
Missing	0 (0)	1 (100)	

^a^Chi-square test

^b^Karnofsky performance status scale

^c^Compared between astrocytoma and other LGG

### Survival analysis

In univariate survival analysis, tumors located in the frontal lobe (*p* = 0.010) and high-dose radiotherapy (*p* = 0.026) were significantly associated with better PFS. Meanwhile, pathology of astrocytoma (*p* = 0.005) and chemotherapy (*p* = 0.024) were associated with worse PFS. For OS, tumors located in the frontal lobe (*p* = 0.009) and high-dose radiotherapy (*p* = 0.015) were significantly good prognostic factors, and pathology of astrocytoma (*p* = 0.037) was still poor prognostic factor (Table 2).

**Table 2:** Univariate analysis of survival outcomes (*n* = 52)

Characteristic	Progression-free survival			Overall survival		
	***p*** value	HR	95% CI	***p*** value	HR	95% CI
Age >40	0.080	2.215	0.909-5.400	0.477	1.412	0.546-3.647
Male	0.379	0.679	0.287-1.609	0.288	0.596	0.230-1.547
Frontal lobe	**0.010**	0.323	0.136-0.767	**0.009**	0.254	0.090-0.714
GTR	0.152	0.505	0.199-1.284	0.293	0.574	0.204-1.613
Astrocytoma	**0.005**	3.524	1.466-8.473	**0.037**	2.781	1.062-7.279
High-dose	**0.026**	0.385	0.165-0.894	**0.015**	0.306	0.119-0.793
Chemotherapy	**0.024**	2.651	1.134-6.194	0.315	1.672	0.614-4.550

*GTR* gross total resection, *HR* hazard ratio, *CI* confidence interval

In multivariate survival analysis, GTR (*p* = 0.010) and pathology of astrocytoma (*p* < 0.001) were independently prognostic factors. Tumors located in the frontal lobe (*p* = 0.016) and high-dose radiotherapy (*p* = 0.028) were independently associated with better OS (Table 3).

**Table 3:** Multivariate analysis of survival outcomes (*n* = 52)

Characteristic	*p* value	HR	95% CI
Progression-free survival			
GTR	0.010	0.259	0.092-0.728
Astrocytoma	< 0.001	6.936	2.465-19.516
Overall survival			
Frontal lobe	0.016	0.274	0.096-0.782
High-dose	0.028	0.335	0.126-0.887

### PFS and OS in relationship to radiation dose

**Fig. 1:**
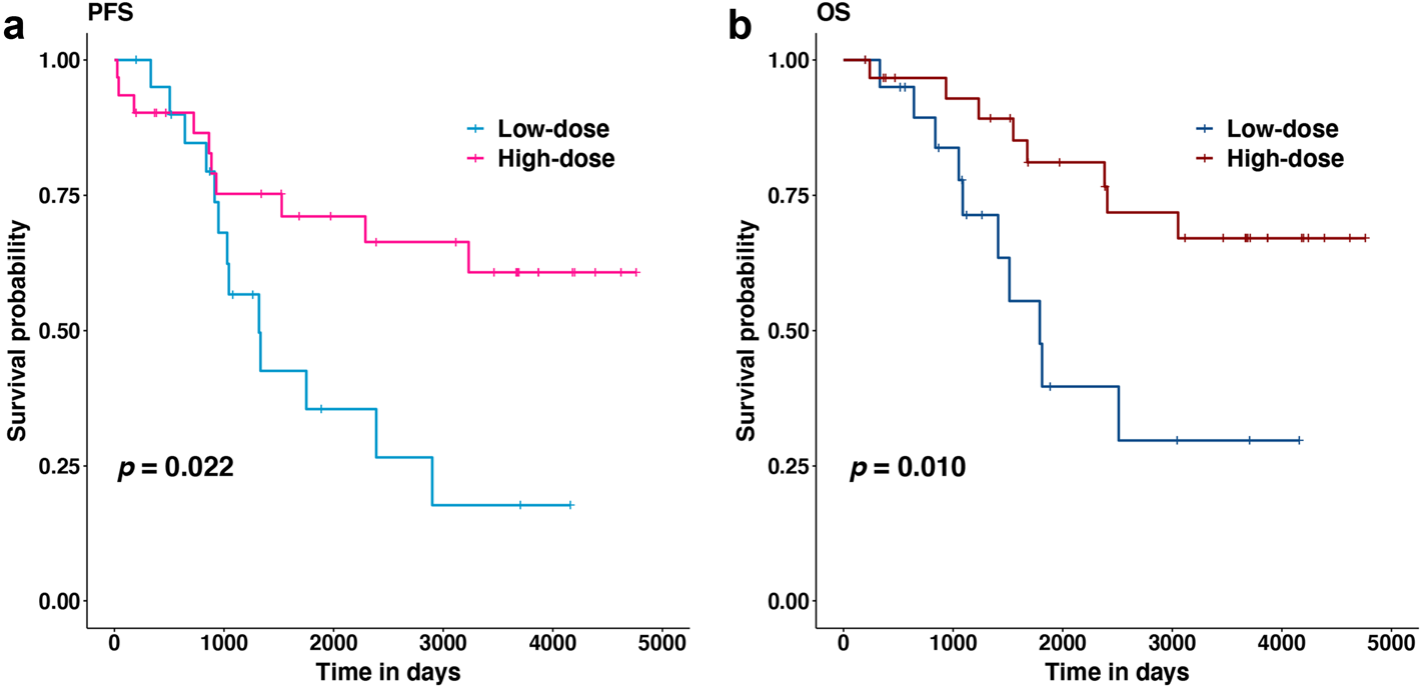
Kaplan-Meier curves of comparison between high-dose and low-dose. **a** progression-free survival (PFS), **b** overall survival (OS)

The PFS and OS of patients treated with high-dose vs. low-dose are shown in Fig. 1a and b. The prognosis of the high-dose group was significantly better (PFS, *p* = 0.022; OS, *p* = 0.010).

## Discussion

As molecular advances have improved the level of diagnosis and outcome prediction for LGG, treatment strategies need to be adjusted according to different molecular subtypes. This study aimed to determine the optimal radiation dose for IDHwt LGG. Survival analysis showed that high-dose radiotherapy independently prolonged patient survival. This finding may help tailor treatment strategies for IDHwt LGG.

The current multidisciplinary treatment strategies for glioma include surgery, radiotherapy, and chemotherapy. In recent decades, these treatments have been developed. Surgical techniques, including intraoperative electrical stimulations [11] and 5-aminolevulinic acid [12] reportedly elevated resection rate. Chemotherapy regimens, such as PCV (procarbazine, lomustine, and vincristine) and temozolomide, significantly improved the survival of gliomas [13, 14]. However, no novel treatment strategies have been proven effective for gliomas. Targeted therapy and immunotherapy have dramatically prolonged the survival of many tumors. For radiotherapy, the application of IMRT and proton therapy has significantly reduced the radiation dose to the surrounding brain tissue. This makes it possible for elevating dose to tumors much safer. However, the effect of higher doses of treatment for LGG patients is still unclear.

Several clinical trials have investigated whether high-dose radiotherapy improved the prognosis of LGG. The EORTC study 22,844 included 379 LGG patients and randomized them between a low-dose arm of 45 Gy and high-dose arm of 59.4 Gy [6]. Meanwhile, the NCCTG study randomized 203 LGG patients between a low-dose arm of 50.4 Gy and high-dose arm of 64.8 Gy [7]. Both studies failed to conclude that LGG patients benefitted from high-dose radiotherapy. This negative result may be attributed to the heterogeneity of LGG, especially across the different molecular subgroups. As a most important biomarker, IDH mutation status deeply influences the pathophysiology of LGG, from survival to therapy response [2, 15]. Tumors with IDH mutations, especially those accompanied by 1p/19q codeletion, may be sensitive to radiotherapy. Thus, a lower dose is sufficient, and complications from higher doses may adversely induce worse prognosis. In contrast, IDHwt LGG is more aggressive, like glioblastoma, and resistant to radiotherapy. In this subgroup, we examined if high-dose was a prognostic factor.

Since 45–54 Gy is the normal recommended dose for LGG [4], we declared doses > 54 Gy as high-dose. Univariate and multivariate survival analyses found that high-dose radiotherapy was significantly associated with better survival in IDHwt LGG. For glioblastoma, the Stupp regimen is the standard treatment [14], and 60 Gy is recommended. In our cohort, 54.4-61.2 (median = 57.6) Gy was administered in the high-dose group. This dose range was deemed reasonable for IDHwt LGG. As cIMPACT-NOW update 3 pointed out that IDHwt LGG carried EGFR amplification, +7/−10 or TERT promoter mutation was considered WHO grade IV [16]. A higher dose, closer to 60 Gy, may bring more survival benefits for these patients.

## Limitations

This study had limitations due to its retrospective nature. First, the radiation field was not evaluated for survival outcomes. Most patients receiving 3D field radiation may reduce bias between groups. Second, the specific chemotherapy regimen and courses for patients were unavailable.

## Conclusions

This study found that high-dose radiotherapy independently improved the survival of IDHwt LGG. This can guide treatments for glioma with known molecular characteristics.

## Data Availability

The datasets generated during and analyzed during the current study are available from the corresponding author on reasonable request.

## Abbreviations

CGGA: Chinese Glioma Genome Atlas
IDH: isocitrate dehydrogenase
IDHwt: IDH-wildtype
GTR: Gross total resection
LGG: low-grade glioma
OS: Overall survival
PFS: Progression-free survival
